# Abnormal Presence of Cholesterol Crystals within Poroma

**DOI:** 10.1016/j.jhsg.2025.100782

**Published:** 2025-08-05

**Authors:** Brant Hannahs, Elizabeth A. Helsper, Ben Van Bockern, Victoria Durkin, Andrew J. Erie, Robert E. Van Demark

**Affiliations:** ∗ University of South Dakota Sanford School of Medicine, Sioux Falls, SD; † Sanford Orthopedics & Sports Medicine – Sanford Health, Sioux Falls, SD; ‡ Department of Graduate Medical Education - University of South Dakota Sanford School of Medicine, Sioux Falls, SD; § Sanford Health, Department of Radiology, Sioux Falls, SD

**Keywords:** Cholesterol crystals, Eccrine poroma, Hand tumor, Poroma

## Abstract

Eccrine poromas are small adnexal neoplasms that arise from the lumen of sweat glands. These lesions are typically small and can mimic other skin conditions, complicating their differentiation during physical examination. We present a case involving a 70-year-old Caucasian man who presented with an unusual mass on his left hand that had considerably increased in size. Initially, a diagnosis of a ganglion was considered; however, subsequent surgical removal and histopathological examination confirmed the diagnosis of poroma. Notably, cholesterol crystals were also found in the mass, a finding that is atypical for this type of neoplasm. If not addressed, poromas may undergo malignant transformation into porocarcinoma, which is associated with poor prognostic outcomes. Furthermore, various systemic or metabolic conditions may influence the development and prognosis of these neoplasms, informing their management and treatment strategies.

A poroma is a benign adnexal tumor that arises from the intraepidermal segment of the sweat gland duct.^1^^,^^2^ It typically affects elderly patients and presents as a solitary, firm papule or nodule.^2^ The origin of poromas was originally thought to be eccrine.^2^ However, research has now produced evidence of cases of poromas from apocrine, sebaceous, and follicular origin.^2^ These masses usually occur on the soles of the feet as well as the head and neck area.^2^ Eccrine poromas rarely occur on the palms, as only eight total cases of this have been reported.^3^ Poromas can be confused with many different types of tumors or skin lesions, and they are usually the same color as the skin.^4^ Although rare, poromas may undergo malignant transformation into porocarcinomas.^5^ A porocarcinoma is differentiated from a benign poroma by the presence of an infiltrative growth pattern and tumor necrosis, as well as increased mitotic activity.^6^ If the mass undergoes rapid growth or causes pain, bleeding, or ulceration, malignant transformation into a porocarcinoma should be considered.^7^ A diagnosis of poromas is dependent on cytology. They are composed of a combination of poroid and cuticular cells, frequently accompanied by duct formation.^5^^,^^8^ Cuticular cells are similar to eccrine duct cuticles, with a prominent eosinophilic cytoplasm.^9^ Poroid cells exhibit prominent round or oval monomorphic nuclei, minimal cytoplasm, and distinct intercellular bridges.^8^ It is important to start with a broad list of differentials because poromas often mimic other tumors and skin lesions.^2^ This can aid in obtaining an accurate diagnosis and improve prognostic outcomes.

## Case Report

A 70-year-old Caucasian man was referred to the hand clinic with the complaint of a painful mass of the left hand that had increased in size over the last 6 months. The mass had been present for many years but had recently grown larger. There was no history of trauma. The patient experienced a sharp, tingling pain in the median nerve distribution when gripping objects, along with mild irritation with gripping. His past medical history was notable for hypertension, hyperlipidemia, coronary artery disease, chronic obstructive pulmonary disease, chronic kidney disease, and benign prostatic hyperplasia. Informed written consent was obtained and the patient agreed to the use of data in publication. The patient was also informed that the participation is completely voluntary, and has the option to withdraw permission at any time without implication.

**Figure 1:**
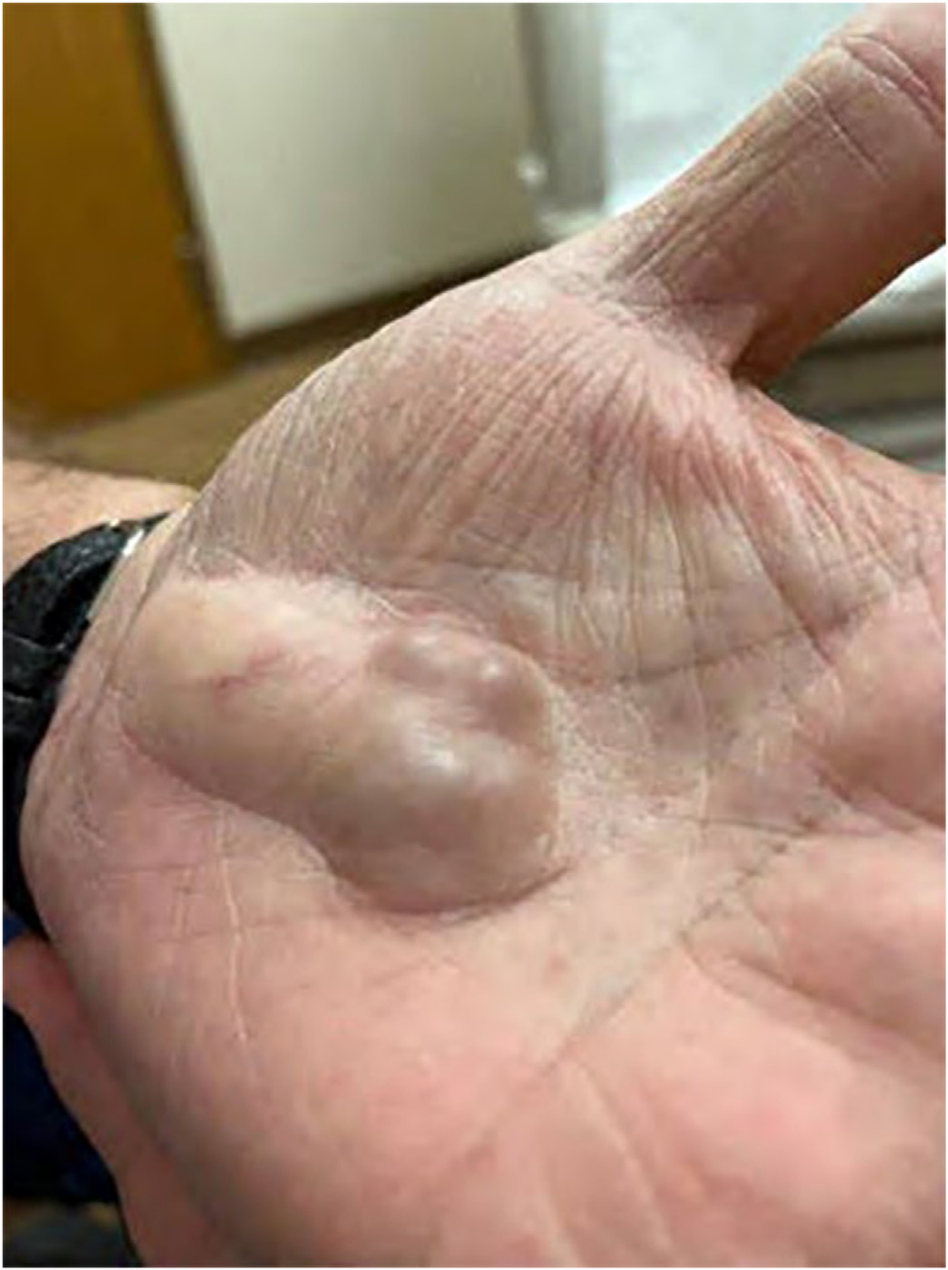
Preoperative clinical photo.

**Figure 2:**
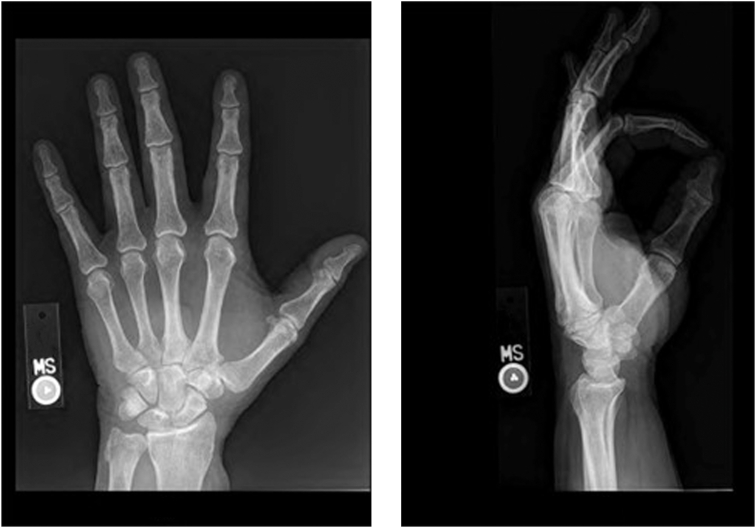
Anteroposterior and lateral left hand radiographs showing minimal degenerative arthritis.

**Figure 3:**
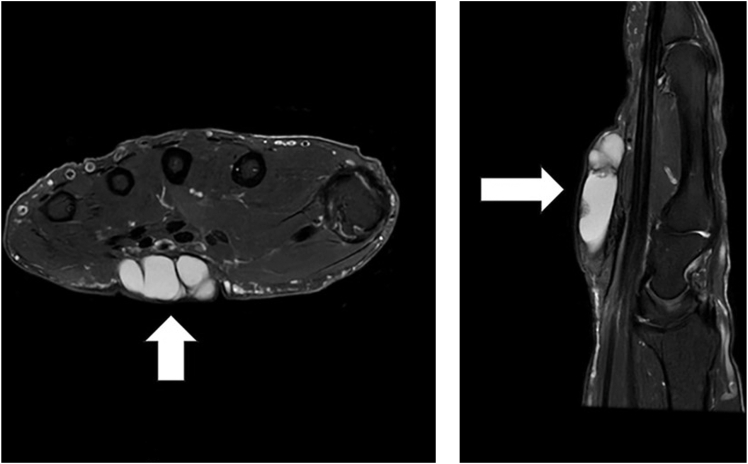
Preoperative MRI demonstrating a multiloculated cystic structure measuring 2.8 cm × 4.7 cm located between the skin and flexor tendon retinaculum (white arrows). Axial T2 image (left). Sagittal T2 image (right).

**Figure 4:**
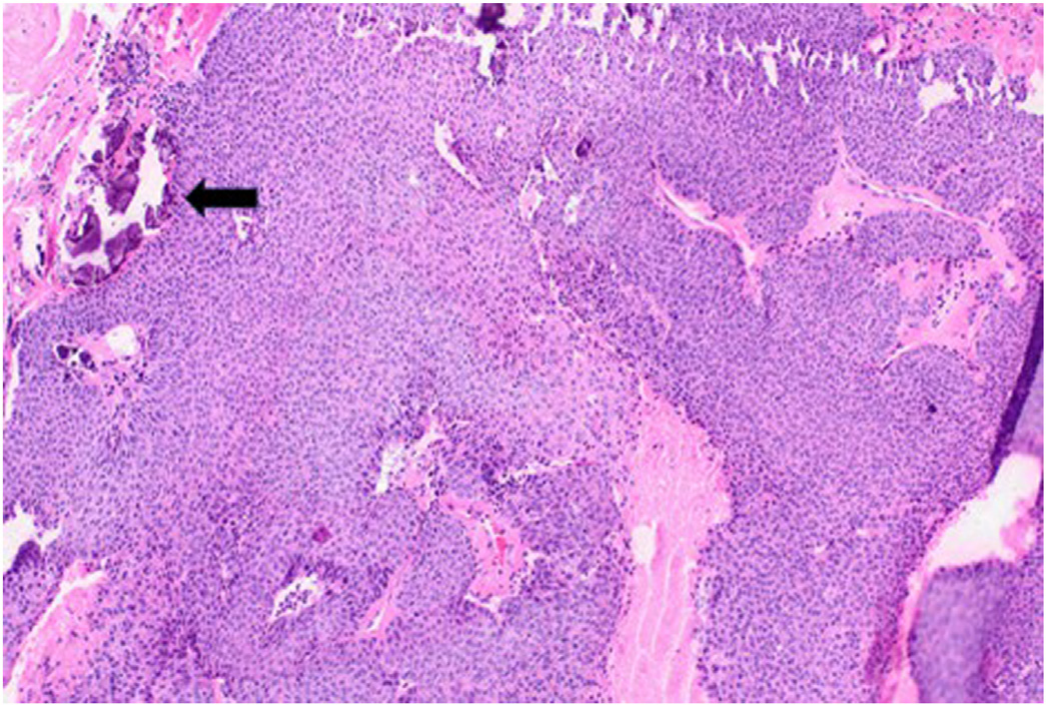
Surgical photomicrograph showing broad anastomosing cords and sheets of bland basaloid to squamoid cells with adjacent calcifications (hematoxylin-eosin, 10×).

**Figure 5:**
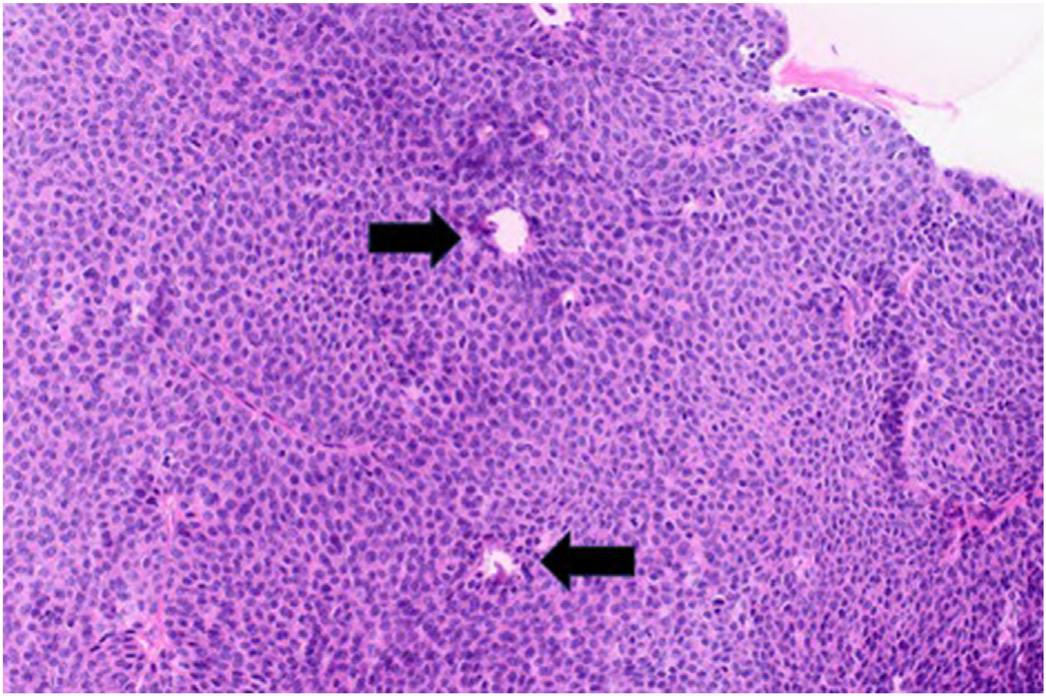
Surgical photomicrograph showing interspersed cuticle-lined ducts (black arrow) within a population of bland basaloid cells (hematoxylin-eosin, 20×).

**Figure 6:**
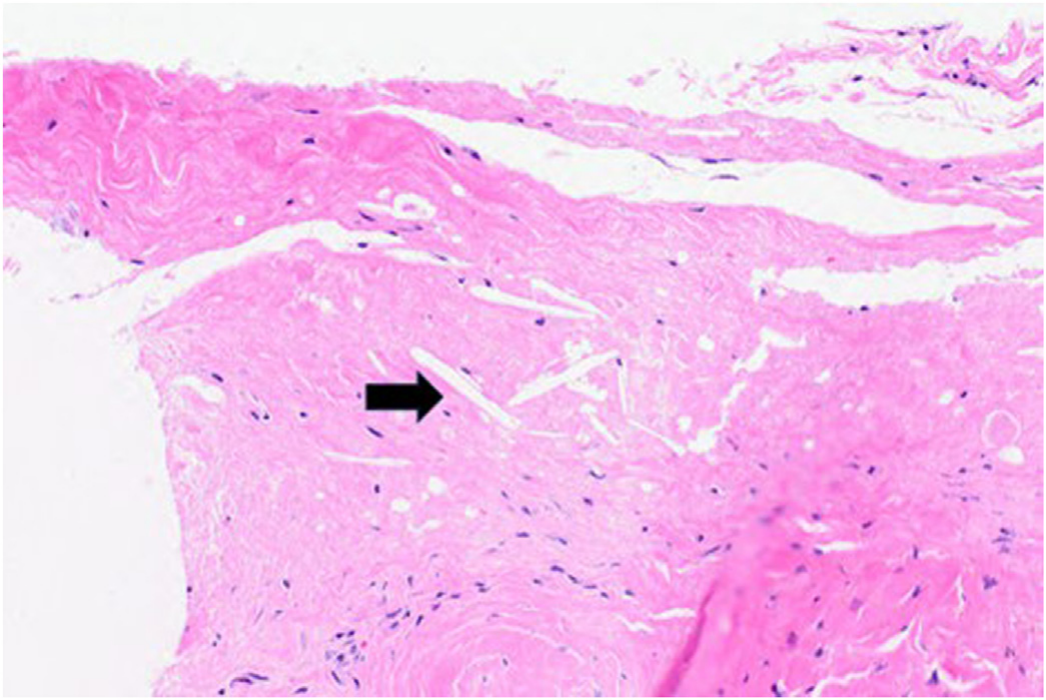
Surgical photomicrograph showing adjacent soft tissue with cholesterol clefts (black arrow) (hematoxylin-eosin, 20×).

**Figure 7:**
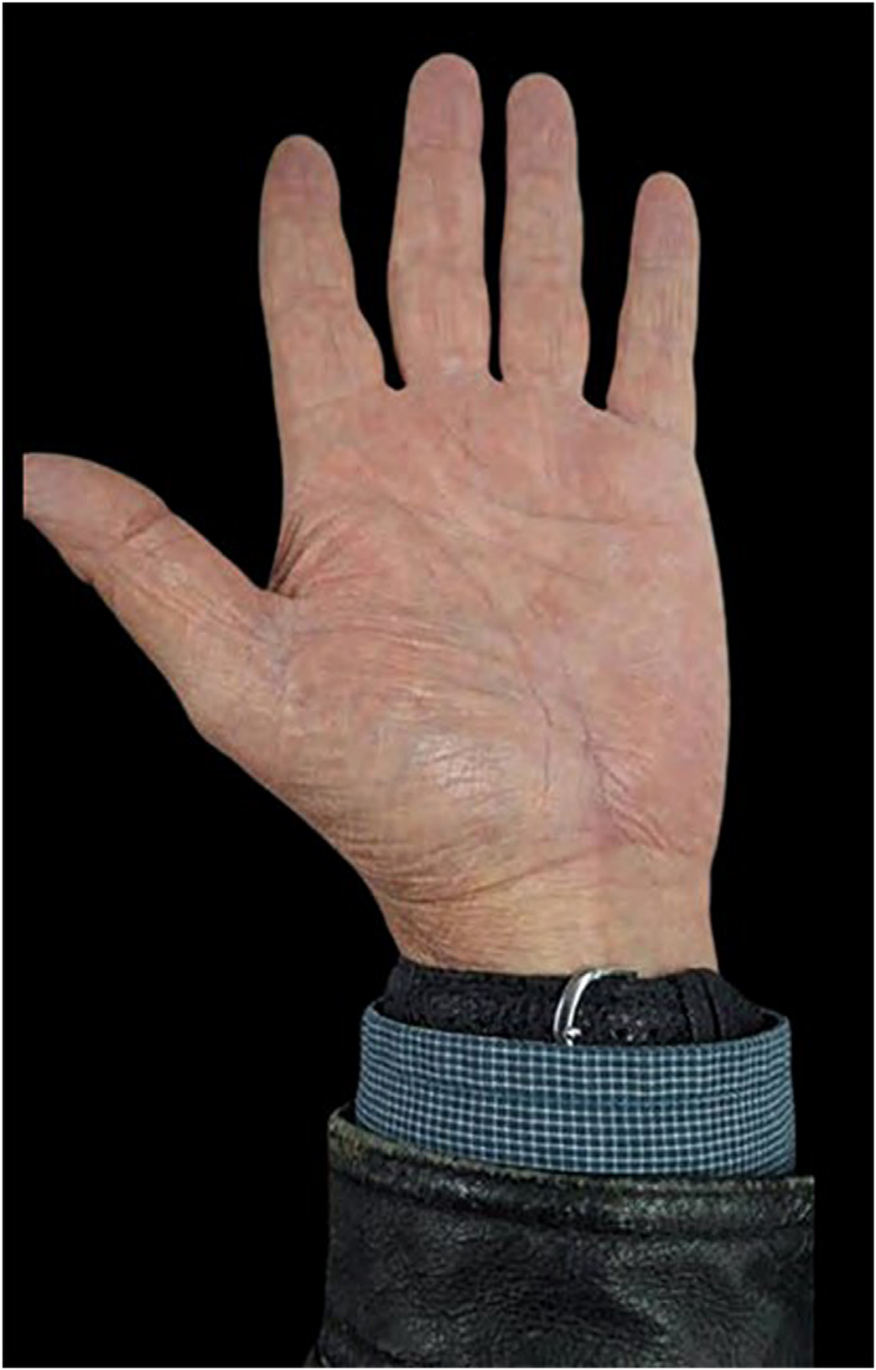
Six-month postoperative photo.

On examination, the mass was nonmobile, compressible, and located over the midportion of his palm (Fig. 1). A blue discoloration was noted, particularly involving the distal aspect of the lesion. There appeared to be a vascular component involving the distal portion of the mass. He also complained of sensory changes in the median nerve distribution. Plain radiographs of the hand (Fig. 2) showed degenerative arthritis. Magnetic resonance imaging (MRI) with and without contrast was performed (Fig. 3). A mass measuring 2.8 cm × 1 cm × 4.7 cm was located between the flexor retinaculum and skin, and MRI demonstrated a small amount of lobular nonenhancing debris that was seen in the lumen of the lesion. A diagnosis of a ganglion was made. Under general anesthesia the mass was excised, and the carpal tunnel was released. Microscopic examination of the mass demonstrated findings consistent with a poroma. These include broad anastomosing cords of sheets of bland basaloid to squamoid cells with adjacent areas of calcification (Fig. 4) and interspersed cuticle-lined ducts within a population of bland basaloid cells (Fig. 5). Unique to this case, there was an unusual feature including cholesterol crystals (Fig. 6). On his first postoperative visit he was doing well with resolution of his preoperative symptoms. Three months after surgery, he was asymptomatic and had no restrictions (Fig. 7). The patient also tested positive for rheumatoid factor and was referred to rheumatology for evaluation of inflammatory arthritis.

## Discussion

The abnormal presence of cholesterol crystals within a poroma in a patient with hyperlipidemia is a noteworthy finding that raises important considerations for both diagnosis and management. In this case, the deposition of cholesterol crystals may result from lipid accumulation, potentially exacerbated by the patient's underlying hyperlipidemia, which predisposes to increased lipid levels in tissues. Of note, there have been no reported cases of cholesterol crystals within poromas, as it is a solid tumor with epithelial proliferation. This association between poromas and hyperlipidemia may not only offer insight into the pathophysiological mechanisms behind crystal formation but also suggests that systemic lipid disorders could influence the characteristics of cutaneous adnexal tumors. The presence of rheumatoid factor further complicates this presentation, indicating possible systemic inflammation.

The possibility of a poroma undergoing malignant transformation is rare. However, it must be ruled out, as eccrine porocarcinoma (EPC) has proven to be highly malignant and to produce poor outcomes. Clinically EPC is extremely variable; however, it commonly appears as an erythematous to violaceous nodule, which may cause itching, pain, ulceration, and even spontaneous bleeding.^6^ However, most cases are asymptomatic, which further complicates the differentiation of EPC from its benign counterpart. Soft tissue biopsy of a suspicious lesion must be performed to differentiate the two entities. EPC is aggressive in nature, with a local recurrence rate of 20%, lymph node involvement of 20%, and solid organ metastasis of 10%.^1^ Wide local excision with 2 mm margins is the recommended approach for the removal of primary EPC, which has been associated with low recurrence rates and increased survival.^6^ Sentinel lymph node biopsy may be warranted if metastatic disease is suspected, although no criteria for lymph node biopsy currently exists.

Although poromas are typically benign, the presence of cholesterol crystals complicates their clinical and histopathological evaluation, as it can resemble conditions associated with lipid or crystal deposition. The challenge lies in distinguishing this rare feature of poromas from other diagnoses. In a series of seven sweat gland tumors of the hand, Patil et al^10^ described the considerable diagnostic challenge these lesions pose given their rarity and resemblance to other skin conditions. They emphasized the value of advances in histopathology, particularly immunohistochemistry, in distinguishing eccrine poromas from other tumors. This supports our case, where a benign tumor could have easily been mistaken for a ganglion.^10^ Additionally, recognizing potential associations between skin lesions and systemic conditions like hyperlipidemia is important, as these comorbidities may play a role in the tumor's development or atypical features. Clinicians should be aware of such rare occurrences, as they may influence the treatment and follow-up strategy for patients.

## Conflicts of Interest

No benefits in any form have been received or will be received related directly to this article.

## References

[1] Sawaya J.L., Khachemoune A. (2014). Poroma: a review of eccrine, apocrine, and malignant forms. Int J Dermatol.

[2] Kyrmanidou E., Fotiadou C., Kemanetzi C. (2023). Eccrine poroma: pathogenesis, new diagnostic tools and association with porocarcinoma-a review. Diagnostics (Basel).

[3] Rasool M.N., Hawary M.B. (2004). Benign eccrine poroma in the palm of the hand. Ann Saudi Med.

[4] Wankhade V., Singh R., Sadhwani V., Kodate P. (2015). Eccrine poroma. Indian Dermatol Online J.

[5] Moore T.O., Orman H.L., Orman S.K., Helm K.F. (2001). Poromas of the head and neck. J Am Acad Dermatol.

[6] Tsiogka A., Koumaki D., Kyriazopoulou M., Liopyris K., Stratigos A., Gregoriou S. (2023). Eccrine porocarcinoma: a review of the literature. Diagnostics (Basel).

[7] Mishra N., Chew D.E.M., Wong K.P.L., Bin Zainuddin M.A. (2024). Eccrine poroma of the palm: a case report and literature review. Cureus.

[8] Kervarrec T., Pissaloux D., Tirode F. (2024). Gene fusions in poroma, porocarcinoma and related adnexal skin tumours: An update. Histopathology.

[9] Deckelbaum S., Touloei K., Shitabata P.K., Sire D.J., Horowitz D. (2014). Eccrine poromatosis: case report and review of the literature. Int J Dermatol.

[10] Patil V.S., Malshikare V.A., Patil A.V. (2024). Sweat gland tumours of the hand: a series of seven cases and review of literature. J Orthop Rep.

